# Modular Synthetic Inverters from Zinc Finger Proteins and Small RNAs

**DOI:** 10.1371/journal.pone.0149483

**Published:** 2016-02-17

**Authors:** Justin Hsia, William J. Holtz, Michel M. Maharbiz, Murat Arcak, Jay D. Keasling

**Affiliations:** 1 Department of Electrical Engineering and Computer Sciences, University of California, Berkeley, California, United States of America; 2 Joint BioEnergy Institute, Emeryville, California, United States of America; 3 Department of Bioengineering, University of California, Berkeley, California, United States of America; 4 Department of Chemical and Biomolecular Engineering, University of California, Berkeley, California, United States of America; 5 Biological Systems & Engineering, Lawrence Berkeley National Laboratory, Berkeley, California, United States of America; University of Illinois at Urbana-Champaign, UNITED STATES

## Abstract

Synthetic zinc finger proteins (ZFPs) can be created to target promoter DNA sequences, repressing transcription. The binding of small RNA (sRNA) to ZFP mRNA creates an ultrasensitive response to generate higher effective Hill coefficients. Here we combined three “off the shelf” ZFPs and three sRNAs to create new modular inverters in *E. coli* and quantify their behavior using induction fold. We found a general ordering of the effects of the ZFPs and sRNAs on induction fold that mostly held true when combining these parts. We then attempted to construct a ring oscillator using our new inverters. Our chosen parts performed insufficiently to create oscillations, but we include future directions for improvement upon our work presented here.

## Introduction

Synthetic biology aims to develop new biological systems and devices, from the modification of existing pathways to the construction of entirely new genetic circuits. Much effort has been spent on the construction of basic elements in bacterial cells such as switches [1, 2], oscillators [3, 4], and logic gates [5–7]. From these basic elements we hope to apply the engineering principles of functional composition to develop more and more complex systems. Constructing *de novo* biological systems has been limited by the number of available parts and the inability to adjust the biological parameters of those parts. Inverters are a key component as they produce negative interactions between components and can act as NOT gates inside engineered pathways. Constructing a large family of tunable inverters would be a significant contribution to the synthetic biology community. An emphasis is placed on orthogonality of parts so that many of these parts may be used in the same cell without altering their behavior. As an application, we then attempted to put three of these inverters together to make a ring oscillator.

### Zinc Finger Protein Technology

ZFPs contain a fold coordinated by a zinc ion and commonly bind DNA, but some are able to bind other molecules such as RNA, proteins, or small molecules. Zinc fingers are frequently found in transcription factors, especially in eukaryotes. There are several classes of zinc finger proteins; the most commonly used class in engineered systems are the C_2_H_2_-type. This type of finger contains a Zn(II) ion coordinated by two cysteine and two histidine residues; a single C_2_H_2_-type zinc finger binds to 3-4 bases of double-stranded DNA. Natural C_2_H_2_-type zinc finger proteins generally contain three or more zinc fingers, allowing them to bind to nine or more base pairs of DNA with dissociation constants commonly in the nanomolar range.

Synthetic ZFPs can be created to target a wide variety of DNA sequences. By targeting a ZFP to bind within a promoter sequence, it is possible to repress transcription by over 250 fold due to steric hindrance of the RNA polymerase [8]. These transcriptional repressing ZFPs act only as DNA binding domains and have no additional activity. By combining ZFP operator sites with nominally constitutive promoters, it is possible to design new promoter-transcriptional repressor pairs. Because of the range of DNA sequences that can be targeted by ZFPs, it becomes possible to create sets of orthogonal promoter-ZFP based transcriptional repressor pairs [8].

### Hybrid sRNA-Repressor Topology

Small RNAs are non-coding RNAs that bind to mRNA and post-transcriptionally regulate the translation of the mRNA [9]. Many sRNAs down regulate the translation of the mRNA they bind to. By constitutively but weakly expressing an sRNA that targets a ZFP-based transcriptional repressor, low levels of ZFP mRNA will not be translated but high levels of ZFP mRNA will result in translation and repression of the cognate promoter [8].

ZFP-based transcriptional repressors described above are monomeric proteins and bind to DNA without any cooperativity (Hill coefficient of 1). We previously showed that in order to construct an oscillator using ZFPs, the binding of sRNA to ZFP mRNA provides an ultrasensitive response in a manner similar to the one produced by protein sequestration in [10] and can be tuned to generate large gains comparable to repressors with higher Hill coefficients. We then showed theoretically that we could use three sets of ZFP-sRNA inverters in a loop to create a ring oscillator [11].

Here we present our experimental work towards constructing a small set of these ZFP-sRNA inverters.

## Analysis

**Fig 1:**
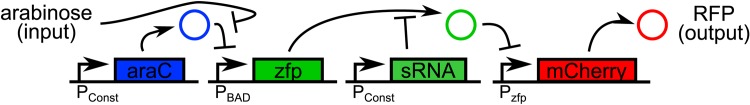
Schematic of the ZFP-sRNA inverters. An inducible promoter is used to vary the production of ZFP, which represses the production of a measured fluorescent protein. The mRNA of the ZFP is targeted by a constitutively-produced sRNA.

A schematic of our ZFP-sRNA inverters is shown in Fig 1. An inducible promoter is used to vary the production of a ZFP, which represses the production of a fluorescent protein. Here we examine the effect of targeting the ZFP mRNA with a constitutive amount of sRNA on our expected experimental results.

We model our inverters using an input of a constant level of ZFP mRNA production, as would be set by our chosen inducer concentration, and using an output of fluorescent protein concentration. We assume that the sRNA-mRNA complexes degrade away at a much faster rate than either individual molecule, eliminating the unbinding reaction from our system of equations. We also use constant terms to represent the production of sRNA from nominally constitutive promoters:

$$\begin{array}{ccc} \frac{d}{dt}s & = & V_{s}N_{s}C-k_{f}sm_{z}-\gamma_{s}s\\ \frac{d}{dt}m_{z} & = & V_{in}-k_{f}sm_{z}-\gamma_{m}m_{z}\\ \frac{d}{dt}p_{z} & = & \epsilon_{z}m_{z}-\gamma_{p}p_{z}\\ \frac{d}{dt}m_{r} & = & VNC(\frac{1}{1+{\left(p_{z}/K\right)}^{n}}+\ell )-\gamma_{m}m_{r}\\ \frac{d}{dt}p_{r} & = & \epsilon_{r}m_{r}-\gamma_{p}p_{r}, \end{array}$$ (1)

where the subscripts *z* and *r* refer to the ZFP and the reporter (or RFP), respectively, and the state variables *s*, *m*, and *p* represent sRNA, mRNA, and protein concentrations. The parameters *V* and *V*_*s*_ are velocity constants, *N* and *N*_*s*_ are copy numbers, *k*_*f*_ is the forward binding rate of sRNA and ZFP mRNA, *K* is the dissociation constant for ZFP-DNA binding, ℓ is the leakage rate normalized to *V*, *γ*_*i*_ are degradation rates, and *ϵ*_*i*_ are the protein translational rates. The parameter *C* is the concentration level generated by a single molecule in an *E. coli* cell.

We compare the steady-state input-output function ${\bar{p}}_{r}\left(V_{in}\right)$ with and without sRNA.

**Without sRNA**:

$$\begin{array}{c} {\bar{p}}_{r,-s}=\frac{\epsilon_{r}}{\gamma_{m}\gamma_{p}}\phi_{n}(\frac{\epsilon_{z}}{\gamma_{m}\gamma_{p}}V_{in}) \end{array}$$ (2)

**With sRNA**:

$$\begin{array}{c} {\bar{p}}_{r,+s}=\frac{\epsilon_{r}}{\gamma_{m}\gamma_{p}}\phi_{n}(\psi (V_{in})) \end{array}$$ (3)

where the functions *ψ*(⋅) and *ϕ*_*n*_(⋅) are defined as follows:

$$\begin{array}{c} \psi \left(x\right)=\frac{\epsilon_{z}}{2\gamma_{m}\gamma_{p}}[\left(x-V_{s}N_{s}C-\frac{\gamma_{s}\gamma_{m}}{k_{f}}\right)+\sqrt{{\left(x-V_{s}N_{s}C-\frac{\gamma_{s}\gamma_{m}}{k_{f}}\right)}^{2}+\frac{4\gamma_{s}\gamma_{m}}{k_{f}}x}\;], \end{array}$$ (4)

$$\begin{array}{c} \text{and}\;\phi_{n}\left(x\right)=VNC(\frac{1}{1+{\left(x/K\right)}^{n}}+\ell ). \end{array}$$ (5)

**Fig 2:**
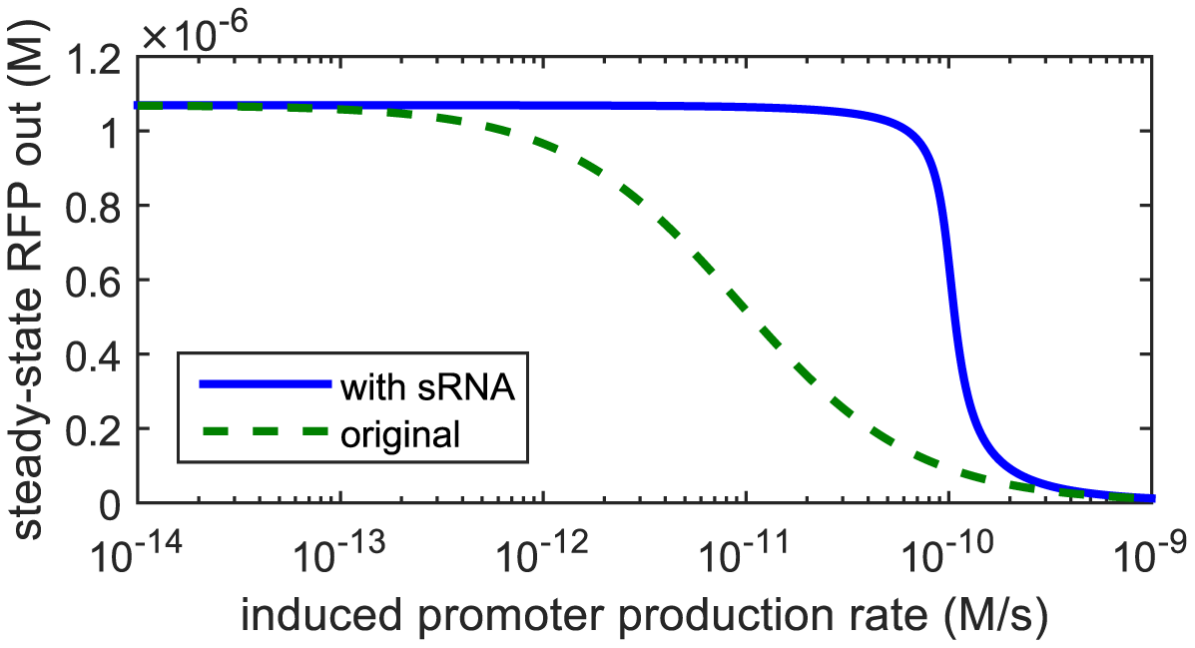
Steady-state input-output map of ZFP inverter showing the effect of adding sRNA to the system. The sRNA thresholding causes the repression to take effect at a higher inducer level and with a sharper slope. The effective Hill coefficient rises from 1 to around 5 with the inclusion of sRNA.

The comparison of these two input-output curves can be seen in Fig 2, where the sRNA thresholding causes the repression to take effect at a higher inducer level and with a sharper slope. The parameters used are *V*_*s*_ = 0.01 s^−1^, *V* = 0.05 s^−1^, *N*_*s*_ = *N* = 10, *C* = 10^−9^ M, *γ*_*s*_ = 2.3 × 10^−3^ s^−1^, *γ*_*m*_ = 1.2 × 10^−2^ s^−1^, *γ*_*p*_ = 3.9 × 10^−4^ s^−1^, *k*_*f*_ = 69 × 10^6^ M^−1^ s^−1^, *K* = 20 × 10^−9^ M, ℓ = 0.001, *ϵ*_*z*_ = *ϵ*_*r*_ = 0.01 s^−1^, and *n* = 1. These parameters all fall within the acceptable ranges given in S1 Table of the Supporting Information of [12]. The effective Hill coefficient rises from 1 to around 5 with the inclusion of sRNA for these parameters.

## Materials and Methods

### Computational

The analytical model was investigated and plotted in MATLAB Version 8.5.0 (R2015a).

Data from experiments were measured using the instruments specified in Experimental Conditions and Procedure and output to files. These files were opened in Microsoft Excel 2013 and the data was analyzed and plotted using standard Excel functions along with a few custom macros. The data sets can be found in S1 Dataset.

### Media and Growth Conditions for Plasmid Construction

*E. coli* liquid cultures were grown at 37°C in lysogeny broth (LB) and colonies were grown on LB-based agar. Antibiotic concentrations of 25 *μ*g/mL for Chloramphenicol (Cm) and 50 *μ*g/mL for Kanamycin (Kan) were used as appropriate.

### Construction of Plasmids

Plasmid construction was done via circular polymerase extension cloning (CPEC) [13] and ’Round-the-horn site-directed mutagenesis [14]. CPEC designs were started by using the j5 DNA assembly design automation software [15] to generate an initial set of oligonucleotides, which were then checked manually and tweaked based on the online Thermo Fisher Scientific Tm Calculator. All polymerase chain reactions (PCRs) were performed using the Phusion Hot Start II High-Fidelity DNA Polymerase (Thermo Scientific F-549).

**Fig 3:**
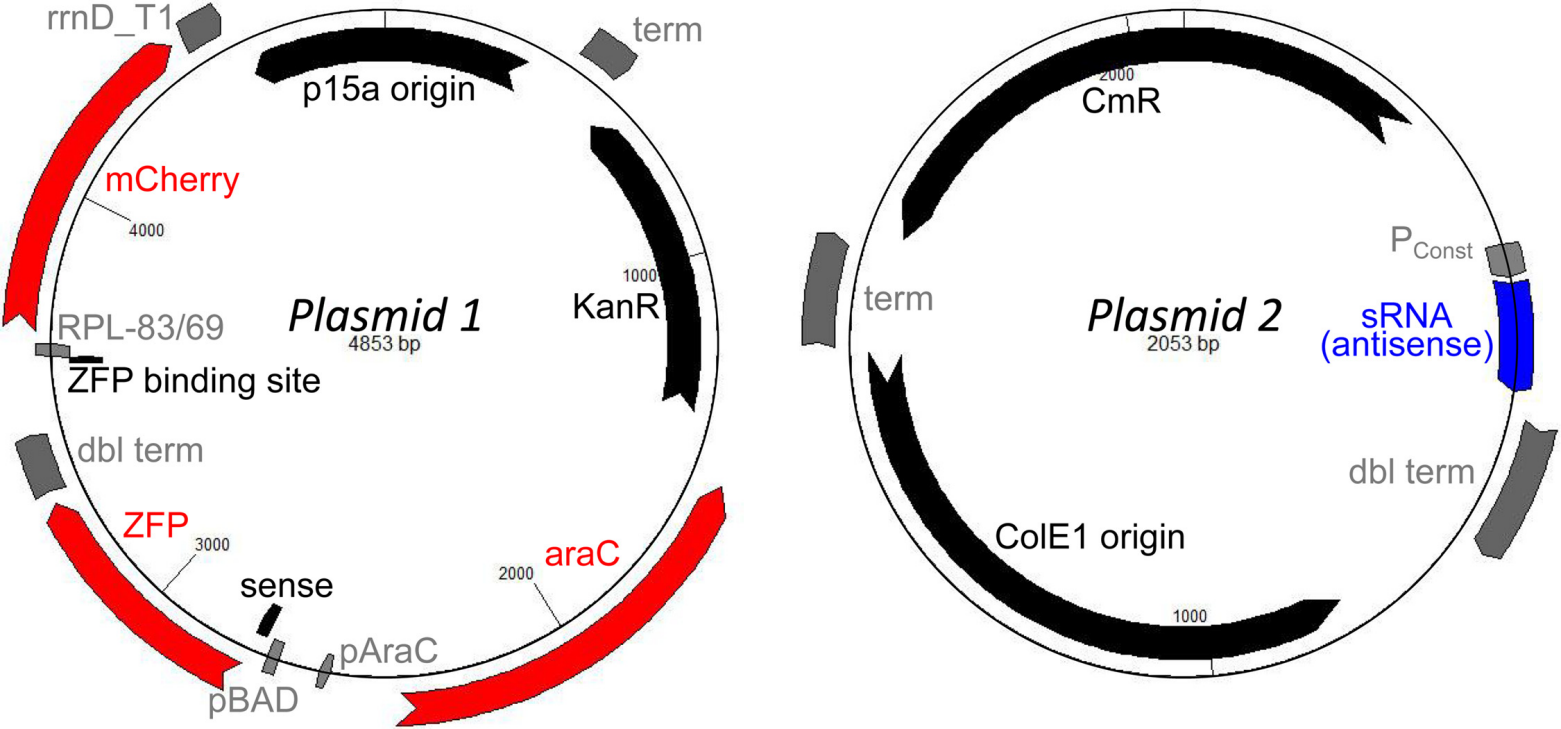
Two-plasmid design for ZFP-sRNA inverters, with a ZFP inverter on Plasmid 1 (left) and the sRNA on Plasmid 2 (right). To build different inverters, we can swap out the ZFP and sRNA, but we must make sure that we also have the proper ZFP binding site, sense region, and output promoter. Experimental strains were made by co-transforming one variant of Plasmid 1 with either a variant of Plasmid 2 or an empty CmR plasmid into DP10 cells [16].

We decided on a two plasmid system in order to separate the ZFP inverter from the sRNA (Fig 3). Given the combinatorial nature of the planned work, this would allow us to do cloning on the two parts of our system independently and then easily combine them as desired. In addition, splitting the plasmids would make cloning more manageable for future experiments involving two or three inverters by reducing the individual plasmid sizes, number of operons, and number of repeated sequences. The list of all plasmids used is found in S1 Table and the oligonucleotides used to make them are given in S2 Table. This section uses nomenclature introduced in Examination of Existing Parts Yields Working Set of Inverter Components below.

#### Construction of ZFP Inverter Plasmids

We started initially with pWH29-77, which already had *P*_*C*_ driving the expression of *araC* and *P*_*BAD*_ driving the expression of ZFP16-59, and added the reporter operon from pWH32-32, which had the ZFP binding site op-3 along with the nominally constitutive promoter RPL-83 driving the expression of mCherry. During this CPEC construction step, we simultaneously added sLS, taken from pWH18-29, to the 5’-UTR of ZFP16-59 and changed the terminator on mCherry, since the same double terminator was used on pWH29-77 and pWH32-32. These were all placed on a p15a/KanR backbone taken from the BglBrick vector pBbA8k-RFP [17]. This initial inverter (pJH4-21) was for ZFP16-59 and sLS and was the basis for all of the other inverters.

The other ZFP inverters were constructed using combinations of the following cloning steps:

1. Swap ZFP16-59 for ZFP16-57 or ZFP16-56 and swap op-3 on RPL-83 to op-18 or op-30 (o4-63 to o4-69).
2. Change RPL-83 to RPL-69 (o9-24, o9-25; must happen after Step 1 to op-18).
3. Swap sLS for s04 or s05 (o6-23, o6-24, o6-37).
4. Perform RBS library search on either sLS (o3-42, o3-43, o9-23) or s04 (o2-14, o7-45, o7-74).

#### Construction of sRNA Plasmids

We had previously constructed plasmids that had aLS constitutively produced by the entire Anderson library [18] of synthetic promoters (20 promoters: pWH39-21 through pWH39-40). These constructs were based on the ColE1/CmR backbone taken from a standard BglBrick vector such as pBbE2c-RFP [17]. Because the difference between a04 and a05 is so small, we first swapped sLS for s04 and then performed mutagenesis to change s04 into s05.

#### Construction of Experimental Plasmids

Final experimental plasmids were obtained by co-transforming a ZFP inverter plasmid variant with either an empty ColE1/CmR plasmid or the corresponding sRNA plasmid variant into DP10 cells [16].

### Experimental Conditions and Procedure

Experiments were run in liquid media using EZ Rich defined medium (Teknova M2105) in 96-well deep well plates (DWPs) with 1.7 mL round wells using an AeraSeal breathable sealing film (Sigma Aldrich A9224) to cover. All liquid culture growth was performed in an INFORS HT Multitron Standard shaker with 25 mm throw at 37°C and 900 rpm. Antibiotic concentrations of 25 *μ*g/mL for Chloramphenicol (Cm) and 50 *μ*g/mL for Kanamycin (Kan) were used.

Each bacterial strain was streaked out from glycerol freezer stock onto double antibiotic LB CmKan agar plates and colonies were grown overnight in a 37°C warm room. Six colonies of each strain were transferred to 400 *μ*L EZ Rich + CmKan wells of a DWP. These cell growth plates were placed on the shaker to grow for about 8 hours.

During growth, experimental DWPs were filled with 392 *μ*L EZ Rich with different induction levels in each of the 12 columns. The chosen induction levels were: 40 mM, 10 mM, 5 mM, 1 mM, 500 *μ*M, 200 *μ*M, 100 *μ*M, 50 *μ*M, 20 *μ*M, 10 *μ*M, 5 *μ*M, and no arabinose. This setup was chosen so that the data for each replicate (all induction levels) came from the same plate and plate-to-plate variation would get averaged out across the replicates. Previous data taken with a single induction level (and all replicates) on each plate produced data with plate-to-plate variability showing up in the induction levels (data not shown).

After removal from the shaker, each column of the growth plates were subcultured at 50X (8 *μ*L) into every column of one of the experimental plates. The experimental plates were then placed on the shaker and grown for about 13 hours.

Upon removal from the shaker, the experimental plates were sampled using a Beckman Coulter Biomek FX laboratory automation workstation and 150 *μ*L from each well was transferred into 96-well black plates with clear flat bottoms (Corning #3631). Bulk fluorescence measurements were taken using a Molecular Devices SpectraMax M2 microplate reader. Settings used were OD measured at 600 nm and RFP measured with 565/620 excitation and emissions wavelengths taken with 30 reads and medium sensitivity flash mode.

In each experimental plate, three sets of controls were used: a positive induction plasmid that has RFP under the control of *P*_*BAD*_ used to measure the input induction level, an “empty” plasmid without RFP, and a “blank” row with just media (no cells). When analyzing the data, background OD levels were calculated by averaging the OD measurement of the “blank” wells for each plate and then subtracted from the OD measurements of the other wells. Next the background fluorescence was calculated by dividing the fluorescence measurements of the “empty” wells by their adjusted OD measurements and averaging for each plate. Final fluorescence values for the wells of interest were calculated by dividing their fluorescence measurements by their adjusted OD measurements and then subtracting off the background fluorescence. Each experiment was run with 6 replicates, average and standard error values were calculated and plotted against the positive induction data in Microsoft Excel.

## Results

### Examination of Existing Parts Yields Working Set of Inverter Components

For our component selection, orthogonality of parts is very important, as large amounts of crosstalk amongst oscillator species make oscillations more difficult to achieve. We also wanted to make our individual inverters as similar in behavior as possible. It is well-known that ring oscillators achieve their largest oscillating parameter region when using identical inverters and in practice it makes input-output matching between inverters easier.

**Fig 4:**
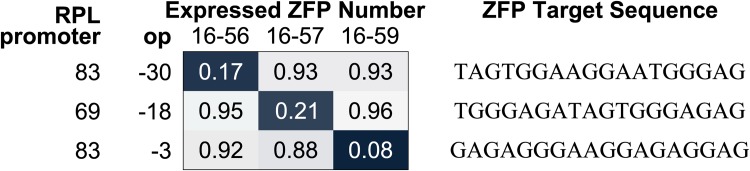
Orthogonal set of 3 promoter-repressor pairs from [8]. Our three chosen ZFP binding sites, op-3, op-18, and op-30, are given with their respective nucleotide recognition sequences and targeting ZFP numbers. Note that op-18 is used on a different BIOFAB promoter than op-3 and op-30.

The ZFPs and operator sites were taken directly from [8], so their nomenclature and numbering are preserved here. We used the ZFP binding sites op-3, op-18, and op-30 and their corresponding ZFP 16-59, ZFP 16-57, and ZFP 16-56 to form a set of orthogonal promoter-repressor pairs (Fig 4). It is important to note that this set of promoter-repressor pairs was built on top of constitutive synthetic promoters from the BIOFAB library [19] and that op-3 and op-30 are placed on BIOFAB RPL-83 while op-18 is placed on BIOFAB RPL-69. Note that these names were taken from the BIOFAB Randomized Promoter Library (RPL) version 1. The most recent BIOFAB promoter library can be found on their data access web service (http://biofab.synberc.org/data/docs/daws?q=data/docs/daws) under “Annotated Parts.” In this database, RPL-83 is named apFAB237, while RPL-69 is not found. For clarity, the promoter sequences used are given in Table 1.

**Table 1:** Synthetic promoters used in ZFP-sRNA inverters. Relative strength numbers found in [18].

Library	Name	Promoter Sequence	Rel Strength
BIOFAB	RPL-69	TTGACAATTAATCATCGGCTCGTAGGGTATGTGGA	n/a
BIOFAB	RPL-83	TTGACAATTAATCATCGGCTCATAACCTTTGTGGA	n/a
Anderson	J23106	TTTACGGCTAGCTCAGTCCTAGGTATAGTGCTAGC	0.47
Anderson	J23108	CTGACAGCTAGCTCAGTCCTAGGTATAATGCTAGC	0.51
Anderson	J23118	TTGACGGCTAGCTCAGTCCTAGGTATTGTGCTAGC	0.56

We investigated the use of three separate sets of sRNAs: one based on the insertion sequence IS10 [20], one based on the plasmid pT181 mechanism [9], and one based on the use of a MicC scaffold [21]. The IS10- and pT181-based sRNAs operate based on sense region and antisense pairings, while the scaffold-based system works with a designable 24 base pair target-binding sequence. Despite designing a scaffold-based sRNA that successfully bound to the mRNA of a reporter RFP, we were unable to design one that bound to one of our ZFPs (data not shown). Additionally, the presence of repeated regions in each individual finger of our ZFPs would have required the addition of a number of synonymous point mutations to produce a set of three orthogonal target regions. A set of five orthogonal IS10-based sense-antisense pairs were identified in [20] and numbered 4, 5, 31, 34, and 49. In our hands, pairs 31, 34, and 49 worked much worse than pairs 4 and 5 to the degree that we were unsure if the sRNA binding was working at all (data not shown). A set of three orthogonal pT181-based sRNAs were presented in [9] and labeled WT, LS, and LS2. The use of these sense regions tended to result in a lower induction fold (i.e. maximum expression level over minimum expression level) of our inverters. Needing just one more sRNA, we proceeded with the best-performing sRNA of the three, which was LS. Note that the 5’-UTR used with a pT181-based sense region includes a RepC minicistron. From here on, we will refer to our chosen set of sense regions as s04, s05, and sLS and our sRNAs (antisenses) as a04, a05, and aLS.

**Fig 5:**
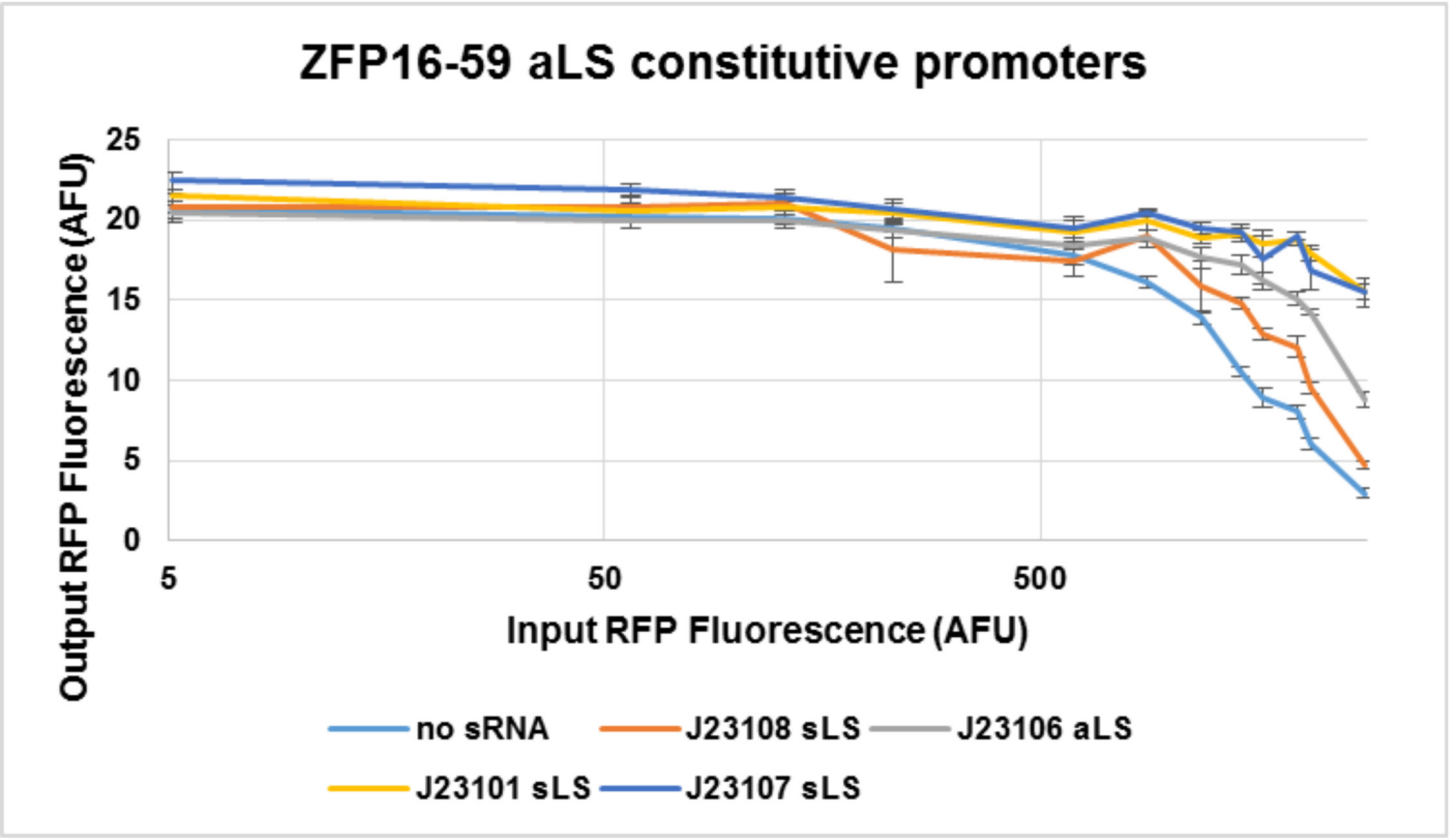
Inverter data showing effect of different constitutive promoters from the Anderson library. More constitutive promoters were tested than are shown here. The magnitude of effect of the sRNA did not match perfectly with the listed relative strengths of the promoters from the parts registry and a number of the promoters resulted in loss of inverting behavior like J23101 and J23107 shown here, likely due to excessive amounts of sRNA in the system.

The sRNAs are produced at a constitutive level, so we looked for three constitutive promoters of roughly equivalent strength. Placing all of the sRNAs on the same operon is also an option, but we decided against it because of polymerase fall off (likely a negligible effect) and the high amount of similarity between sRNAs from the same set (e.g. a04 and a05). Placing them onto separate promoters allows us to space them out and place them in opposing directions as needed. We chose the promoters J23106, J23108, and J23118 from the Anderson library of synthetic promoters (see Table 1). We tested the promoters from the library with listed relative strengths close to J23108, but found that many provided undesired behavior (see Fig 5). Altering the promoter strength (and/or copy number) of the sRNAs would change the degree of the effect we expect to see. We were looking for an intermediate level where the thresholding effect of the sRNAs would be clearly visible without becoming so sharp that we would miss the repression effect with our finite number of inducer levels, especially since induction is never quite linear.

We investigated small RBS libraries to use with the different sense regions in order to try to maximize inverter induction fold without affecting sRNA binding significantly. For the IS10-based sense regions, we looked at a two-base pair library in the location of the mutated Shine-Dalgarno region identified in [20] and identified TC as the optimal RBS. For the pT181-based sense regions, we looked at a library of 256 using the nucleotide sequence “RRRRNN” starting 14 bp before the beginning of the ZFP start codon, where R stands for A or G, and N is any nucleotide. The best we found was AAAGGA.

We planned from the start on using *P*_*BAD*_ as our inducible promoter with arabinose. We did test swapping in *P*_*LtetO*-1_ and *P*_*LlacO*-1_ as well, but did not see any significant improvements (data not shown). To eliminate the “all-or-none” behavior of the *P*_*BAD*_ promoter, we had to select an experimental strain of *E. coli* that expresses *araE* [22]. We used the DP10 strain from the Keasling lab [16].

For our output protein, we chose mCherry because it is monomeric, matures quickly, and is photostable [23]. mCherry was placed on the synthetic promoter-ZFP binding site pairings developed in [8], so we used a very strong 5’-UTR in the Bujard RBS [24].

### Inverter Data Shows Desired Qualitative Behavior and General Trends of Modular Components

**Fig 6:**
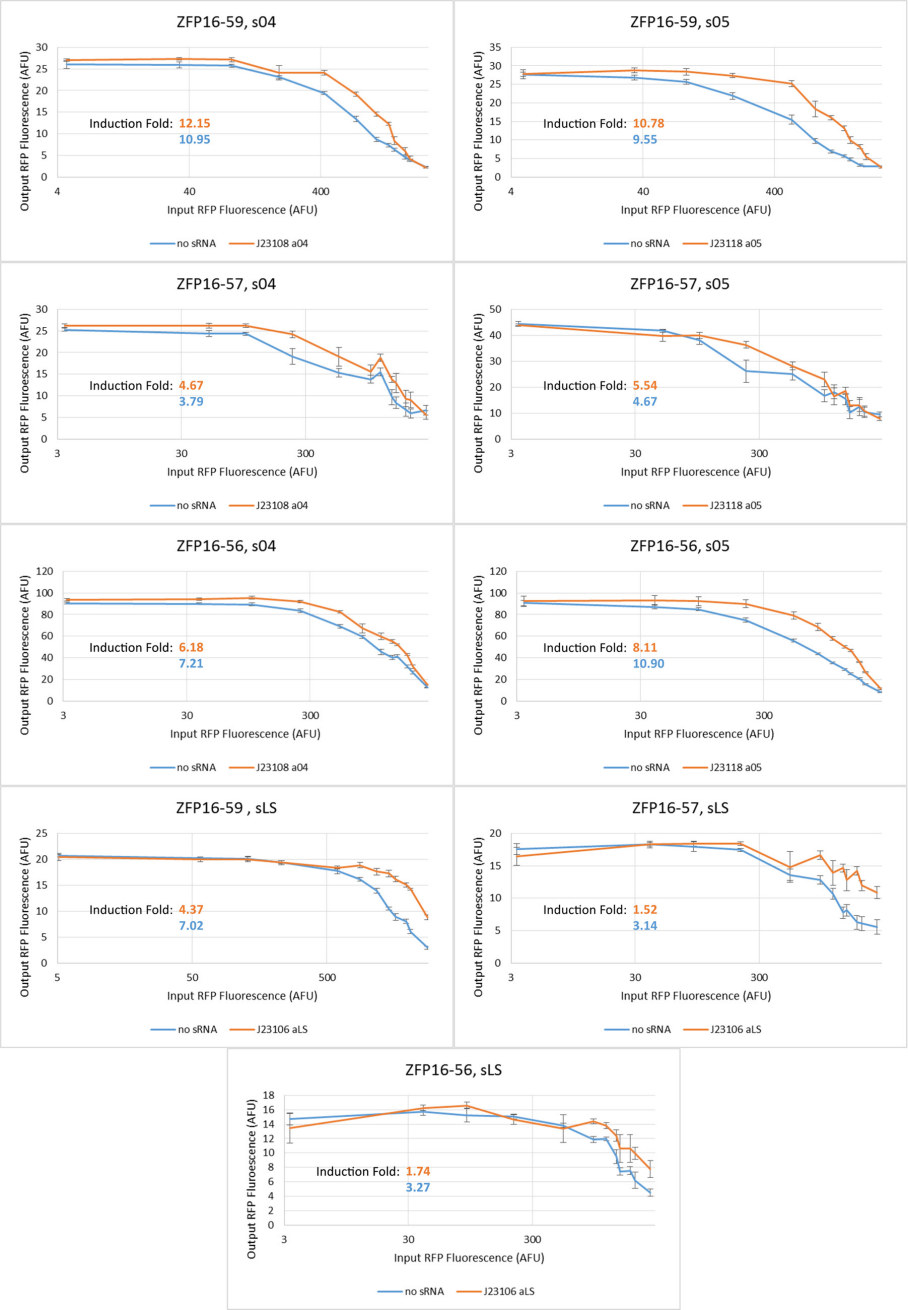
Inverter data for 3 ZFPs and 3 sRNAs in combination with induction folds.

**Fig 7:**
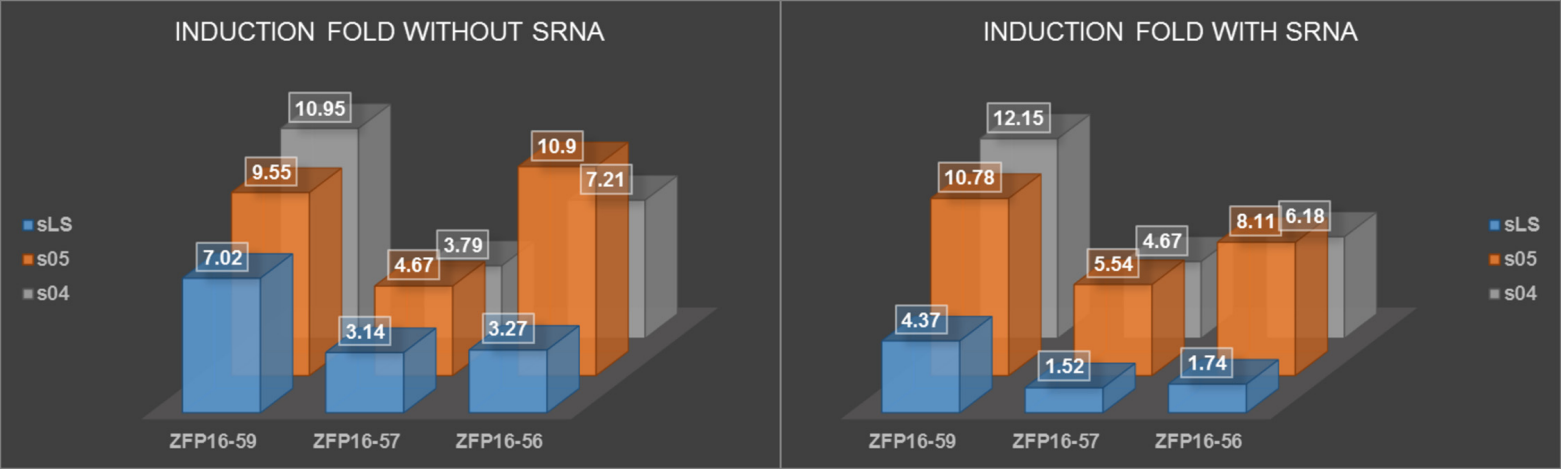
Grid visualization of induction folds of our inverter data from Fig 6. Induction fold is one indicator of how well our inverter is working since it indicates how significant the difference between “on” and “off” states are.

Inverter steady-state input-output functions are shown in Fig 6. Data for nine different inverters is shown, each inverter with one of three ZFPs paired with one of three sRNA sense regions, as well as the induction fold (maximum output/minimum output). Each plot corresponds to one of these inverters in the presence and absence of a constitutive amount of the corresponding sRNA. The induction fold numbers are visualized in Fig 7.

Inverter orthogonality tests were not run because the orthogonality of the individual components were demonstrated in previous work [8, 9, 20]. Using multiple inverters together would also demonstrate viable levels of orthogonality.

### Inverters Prove Insufficient to Construct Ring Oscillator

**Fig 8:**
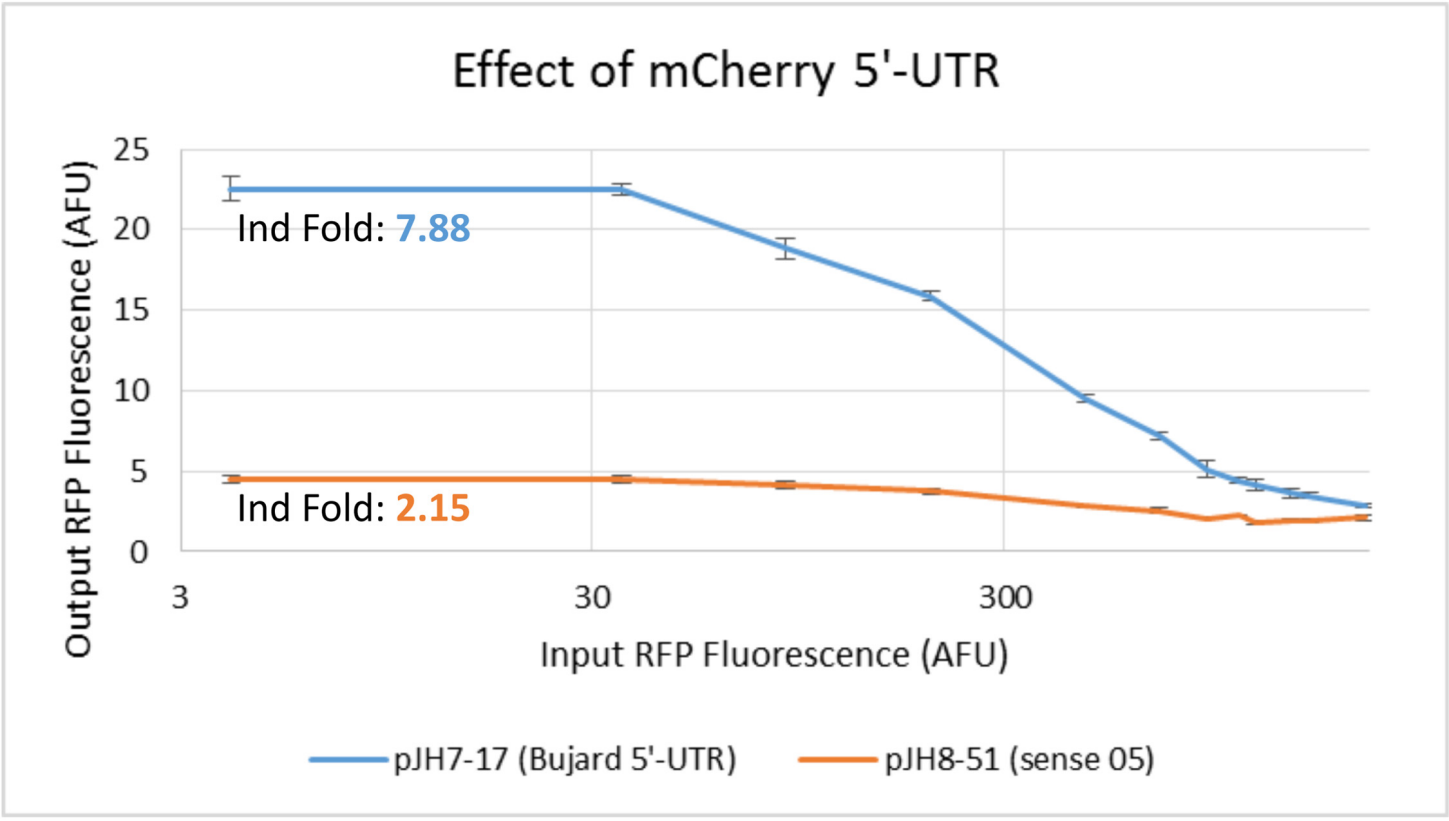
Inverter data showing effect of sense region 5’-UTR. Swapping the Bujard 5’-UTR on the output RFP with one including an IS10-based sense region drastically reduces the induction fold from 7.88 to 2.15.

We also tested whether we could put two of our inverters in series to produce a delayed buffer effect as an intermediate step to putting together a ring oscillator. The first test was to introduce a sense region in the 5’-UTR on our reporter. Unfortunately we saw that this greatly reduced our induction fold (Fig 8). Based on this evidence, we concluded that with our current set of parts two inverters in series was unlikely to work due to inverter input-output mismatch.

## Discussion

### Inverter Performance

In order to put a three-component ring oscillator together, we selected three ZFPs and three sRNAs and demonstrated their modularity by running steady-state fluorescence experiments on all nine different combinations. Individually, every ZFP-sRNA inverter did what it was supposed to do: produce “high” output at “low” input levels and “low” output at “high” input levels. Additionally, the expected qualitative change in behavior with the addition of a constitutive amount of sRNA in the system was demonstrated (compare Figs 2 to 6).

The highest induction fold achieved was around 12 (91% repression), which is enough to be useful depending on the application and compares favorably to the ZFP repressors in isolation (see Fig 4 and [8]). While there are better-performing repressors available such as *P*_*LtetO*-1_, *P*_*LlacO*-1_, and *P*_*lac*/*ara*-1_, which have induction folds >600 [24], ZFPs add to the limited library of promoter-repressor pairs. In addition, the modularity of ZFPs allow us to target promoters that do not have known repressors, such as the growing library of synthetic constitutive promoters.

The goals of building ZFP-sRNA inverters were to develop a “large” library of orthogonal parts with nearly-identical parameters, as these would aid in the construction of a new synthetic ring oscillator. While neither goal was quite achieved and the ring oscillator has yet to come together, there is much to be learned from the effort. In an attempt to avoid revisiting the design work on the ZFPs, we investigated the minimal number of parts needed for our ring oscillator. Even then, the parts chosen displayed an unexpected amount of variation in behavior.

In Fig 7, induction fold provides a loose measure of how well the inverters work as it is an indicator of how different the “on” and “off” states are. Another important metric would be the slope of the linear region of the inverter input-output map, but given the limited number of induction levels used, this value is difficult to determine accurately from the data.

In terms of effect on induction fold, the ZFPs were generally ordered with ZFP16-59 performing the best and ZFP16-57 performing the worst. ZFP16-56 did seem to work surprisingly well with s05. s04 and s05 performed comparably in regards to induction fold and sLS performed significantly worse. Additionally, the data with sLS and aLS suffered in induction fold because the output was unexpectedly high at maximal induction (whereas s04 and s05 achieved nearly the same minimum value with and without sRNA present). Based on the data of different constitutive promoters (Fig 5), it is more likely that this was caused by the choice of J23106. As mentioned previously, the Anderson library of constitutive promoters did not exhibit the expected ordering of relative promoter strengths as provided [18]. These trends were generally consistent and the worst induction fold was achieved with the combination of ZFP16-57 and aLS, as expected.

Attempting to comment on the possible sources of these differences is difficult, since the induction fold is affected by nearly every interaction in our inverter. The most consistent trend is the ordering of the ZFPs in that, with one exception, using ZFP16-59 outperformed ZFP16-56, which in turn outperformed ZFP16-57. This would suggest a general ordering of the binding affinity and repressive strengths of the ZFPs to their targeted promoters, but we can’t draw any strong conclusions due to our small data set and our inability to differentiate between the two effects.

### Ring Oscillator

There are two main reasons that putting two inverters in series was unsuccessful. The first is the low induction fold of each individual inverter and the second is the input-output mismatching between inverters. Each inverter was run with RFP having the Bujard 5’-UTR, which is known to be very strong. However, once multiple inverters are used in series, the intermediate operons must have sense regions as well. When we measured the effect of adding this sense region (Fig 8), there was a drastic reduction in promoter output range, which would affect the intermediate ZFP variations in concentration.

Secondly, the inverting behavior is seen in the very top portion of the induction range. In order for the ring oscillator to work, we need the steady-state solution to lie in the linear regime of the input-output map, so that changes in the input level around the steady state cause significant changes in the output as well. Since the linear regime is in the upper input range, we would need each inverter to be able to produce a very large maximal amount of ZFP, which is unlikely given the effect of the sense region on the promoter. The sense region further compounds the problem by reducing the inverter induction fold, meaning that the input changes passed to the next inverter are of small magnitude.

### Future Work

The ring oscillator should work by either selecting better parts or improving the current ones. The chief components in question are the promoter-repressor pairs. If we decide to continue working with ZFPs in *E. coli*, a few of the different improvements to investigate include:

- Changing the number of fingers used in each ZFP and trying different target sequences.
- Using multiple copies of the ZFP binding site on a promoter.
- Using known strong promoters (e.g. *P*_*LtetO*-1_) or some of the many others contained in the BIOFAB promoter library. This would provide us with greater induction fold, although we would eventually want three promoters of roughly equivalent strength.
- Looking for a better-performing set of orthogonal ZFPs.

ZFPs are not the only option for designable repressors [25]. All that is needed is something that will bind to the DNA adjacent to a promoter to block transcription. The main alternatives are CRISPRi [26] from recent work on clustered regularly interspaced short palindromic repeats (CRISPR) [27] and transcription activator-like effectors (TALEs) [28], both of which allow binding to an arbitrarily specified nucleotide sequence. TALEs are similar to ZFPs in that they are constructed from a sequence of repeats where two amino acids called the repeat-variable diresidues (RVDs) specify a single base that will be bound. So for binding the same sequence, a TALE will contain about three times as many repeated sequences as a ZFP. In CRISPRi, one coexpresses a catalytically dead CRISPR-associated system protein (dCAS9) with designable guide RNAs to form DNA recognition complexes. ZFPs are the most difficult to design and construct properly but bind the most specifically. CRISPR systems are the easiest to design (only need to change the guide RNA), but suffer from off-target effects, which are mitigated in CRISPRi by the lack of endonuclease cutting activity. TALEs fall in the middle in terms of ease of design and construction and binding specificity.

## Supporting Information

S1 Table Plasmids used in ZFP-sRNA inverter experiments. Intermediate cloning plasmids first, followed by the final experimental plasmids. All plasmids were transformed into the strain DP10 [16].
(PDF)

S2 Table Oligonucleotides used in the construction of the ZFP-sRNA inverter plasmids. Here “for” denotes a forward primer, “rev” denotes a reverse primer, and “RTH” indicates use in ’Round-the-horn site-directed mutagenesis. Bases highlighted in red indicate the specific regions being changed.
(PDF)

S1 Dataset Dataset for ZFP-sRNA inverters. ZIP file containing Excel 2013 files of raw and adjusted data presented in this paper.
(ZIP)
