# Supplementary material for: Modular Synthetic Inverters from Zinc Finger Proteins and Small RNAs
Source: PLoS One. 2016 Feb 17;11(2):e0149483. doi: 10.1371/journal.pone.0149483 (PMC4757538; doi:10.1371/journal.pone.0149483)
Supplement: S1 Table — Intermediate cloning plasmids first, followed by the final experimental plasmids. All plasmids were transformed into the strain DP10 [16]. (PDF) [file pone.0149483.s001.pdf]

S1 Table. Plasmids used in ZFP-sRNA inverter experiments. Intermediate cloning plasmids first, followed by the final experimental plasmids. All plasmids were transformed into the strain DP10 [1].

Name	Resistance	Origin	Description
pWH29-77	CmR	SC101**	AraC on P_C and ZFP16-59 on P_{BAD}.
pWH32-32	KanR	ColE1	mCherry on RPL-83 with op-3.
pWH18-29	CmR	p15a	GFP on pJ23119 with sLS. Used to get sLS.
pJH4-21	KanR	p15a	Operons from pWH29-77 and pWH32-32 moved onto KanR/p15a vector with sLS added to ZFP16-59 and mCherry terminator changed. The basis for the ZFP inverter plasmid.
pJH5-57	KanR	p15a	pJH4-21 with sLS changed to s04.
pWH16-57	AmpR	ColE1	Plasmid from William Holtz with ZFP16-57.
pWH16-56	AmpR	ColE1	Plasmid from William Holtz with ZFP16-56.
pWH39-21<br>pWH39-40	CmR	ColE1	Twenty plasmids with aLS on numerically-ordered promoters J23100 to J23119. The bases for the sRNA plasmids.
VKM40	AmpR	ColE1	Plasmid from Vivek Mutalik with a04 on $P_{LlacO\text{-}1}$ and LacI. Used to get a04; a05 made from a04 via cloning.
pJH1-81	KanR	p15a	Empty plasmid.
pWH17-39	CmR	ColE1	Empty plasmid.
pJH4-37	KanR	p15a	AraC on P_C and mRFP1 on P_{BAD} for input measurement. Originally pBbA8k-RFP [2].
pJH7-17	KanR	p15a	ZFP inverter plasmid with ZFP16-59, op-3, RPL-83, s04.
pJH7-59	KanR	p15a	ZFP inverter plasmid with ZFP16-59, op-3, RPL-83, s05.
pJH4-40	KanR	p15a	ZFP inverter plasmid with ZFP16-59, op-3, RPL-83, sLS.
pJH9-55	KanR	p15a	ZFP inverter plasmid with ZFP16-57, op-18, RPL-69, s04.
pJH9-56	KanR	p15a	ZFP inverter plasmid with ZFP16-57, op-18, RPL-69, s05.
pJH9-52	KanR	p15a	ZFP inverter plasmid with ZFP16-57, op-18, RPL-69, sLS.
pJH7-57	KanR	p15a	ZFP inverter plasmid with ZFP16-56, op-30, RPL-83, s04.
pJH7-60	KanR	p15a	ZFP inverter plasmid with ZFP16-56, op-30, RPL-83, s05.
pJH4-64	KanR	p15a	ZFP inverter plasmid with ZFP16-56, op-30, RPL-83, sLS.
pJH5-56	CmR	ColE1	sRNA plasmid with J23108 and a04.
pJH6-26	CmR	ColE1	sRNA plasmid with J23118 and a05.
pWH39-27	CmR	ColE1	sRNA plasmid with J23106 and aLS.
pJH6-63	KanR	p15a	Two ZFP inverter plasmid with ZFP16-56, op-30, RPL-83, s04 targeting ZFP16-59, op-3, RPL-83, s05.
pJH7-42	CmR	ColE1	Two sRNA plasmid with a04 on J23108 and a05 on J23118.
pJH8-51	KanR	p15a	Plasmid 1* with ZFP16-59, op-3, RPL-83, s04/s05.
pJH7-61	KanR	p15a	Plasmid 3* with ZFP16-56, op-30, RPL-83, s04 targeting mCherry, s05.

References

1. Kizer L, Pitera DJ, Pfleger BF, Keasling JD. Application of Functional Genomics to Pathway Optimization for Increased Isoprenoid Production. Applied and Environmental Microbiology. 2008;74(10):3229–3241.

2. Lee TS, Krupa RA, Zhang F, Hajimorad M, Holtz WJ, Prasad N, et al. BglBrick vectors and datasheets: A synthetic biology platform for gene expression. Journal of Biological Engineering. 2011;5(1):1–14.
