# Supplementary material for: Modular Synthetic Inverters from Zinc Finger Proteins and Small RNAs
Source: PLoS One. 2016 Feb 17;11(2):e0149483. doi: 10.1371/journal.pone.0149483 (PMC4757538; doi:10.1371/journal.pone.0149483)
Supplement: S2 Table — Here “for” denotes a forward primer, “rev” denotes a reverse primer, and “RTH” indicates use in ’Round-the-horn site-directed mutagenesis. Bases highlighted in red indicate the specific regions being changed. (PDF) [file pone.0149483.s002.pdf]

S2 Table. Oligonucleotides used in the construction of the ZFP-sRNA inverter plasmids. Here "for" denotes a forward primer, "rev" denotes a reverse primer, and "RTH" indicates use in 'Round-the-horn site-directed mutagenesis. Bases highlighted in bold font indicate the specific regions being changed.

**Name**	**Description**	**Sequence**
o4-63	ZFP swap pJH4-40vec for (to op-18)	cctatcagctgcgtgctttctat**tgggagatagtgggagag**ttgacaattaatcatcgg
o4-69	ZFP swap pJH4-40vec for (to op-30)	cctatcagctgcgtgctttctat**tagtggaaggaatgggag**ttgacaattaatcatcgg
o4-64	ZFP swap pJH4-40vec rev (RBS AAAGGA)	cctggttccagcatagatcctatcctttagatc
o4-65	ZFP pWH16-56/57 for	tagccggttgtaaggatctaaaggataggatctatgctggaaccaggatc
o4-66	ZFP pWH16-56/57 rev	gcctggagatccttactcgagtttggatccttattaagaggttttagatc
o4-67	ZFP swap pJH4-40 term spacer for	ggatccaaactcgagtaaggatctccaggc
o4-68	ZFP swap pJH4-40 term spacer rev	atagaaagcacgcagctgatagggtcga
o7-45	RTH s04/s05 RBS for	**nn**agacaacaagatgtgcgaactcgatgctggaac
o2-14	RTH s04 RBS rev	ttattgatttttggcatggagaaacagtagag
o7-74	RTH s05 RBS rev	ttattgattttacgcatggagaaacagtagag
o3-42	vec sLS RBS lib rev	agatccttacaaccggctattagagtagc
o3-43	vec sLS RBS lib for	aggatctatgctggaaccaggatc
o9-23	sLS RBS lib	gatcctggttccagcatagatccta**nnyyyy**agatccttacaaccggctattagagtagc
o7-24	RTH op rev	atagaaagcacgcagctgatagggtcga
o7-25	RTH op-3 for (RPL-83)	**gagagggaaggagaggag**ttgacaattaatcatcggctcataacc
o7-81	RTH op-18 for (RPL-83)	**tgggagatagtgggagag**ttgacaattaatcatcggctcataacc
o7-80	RTH op-30 for (RPL-83)	**tagtggaaggaatgggag**ttgacaattaatcatcggctcataacc
o9-24	RTH RPL-83 to 69 for	tgtggaacaattcattaaagaggagaaaggtac
o9-25	RTH RPL-83 to 69 rev (op-18)	taccctacgagccgatgattaattgtcaactctcc
o6-19	vec pWH39-29 for	ggatcctaactcgagtaaggatctccaggca
o6-20	vec pWH39-29 rev (pJ23108)	gctagcattatacctaggactgagctagctgtcaga
o6-21	a04 from VKM40 for	agctcagtcctaggtataatgctagctcgcacatcttgttgtctgatta
o6-22	a04 from VKM40 rev	gagatccttactcgagttaggatccctgatgaatcccctaatgattttg
o6-34	RTH a04/a05 rev	aaatcaataatcagacaacaagatgtgcga
o6-35	RTH a05 for	**tacg**cgaaaccatttgatcatatgacaagatgtg
o1-6	RTH to pJ23118 for	cggctagctcagtcctaggtattgtgctagctcgcacatcttgttgtctg
o6-49	RTH to pJ23118 rev	tcaaacgtgccagatctttagaattcgatatctg
o6-23	RTH to s04/s05 for	caacaagatgtgcgaactcgatgctggaaccaggatc
o6-24	RTH to s04 rev (RBS TC)	tct**ga**ttattgattttt**tg**gcatggagaaacagtagagagttgc
o6-37	RTH to s05 rev (RBS TC)	tct**ga**ttattgatttt**ac**gcatggagaaacagtagagagttgc
